# Effects of Mutations and Ligands on the Thermostability of the l-Arginine/Agmatine Antiporter AdiC and Deduced Insights into Ligand-Binding of Human l-Type Amino Acid Transporters

**DOI:** 10.3390/ijms19030918

**Published:** 2018-03-20

**Authors:** Hüseyin Ilgü, Jean-Marc Jeckelmann, Claire Colas, Zöhre Ucurum, Avner Schlessinger, Dimitrios Fotiadis

**Affiliations:** 1 Institute of Biochemistry and Molecular Medicine, and Swiss National Centre of Competence in Research (NCCR) TransCure, University of Bern, CH-3012 Bern, Switzerland; hueseyin.ilgue@ibmm.unibe.ch (H.I.); jean-marc.jeckelmann@ibmm.unibe.ch (J.-M.J.); zoehre.ucurum@ibmm.unibe.ch (Z.U.); 2 Department of Pharmacological Sciences, Icahn School of Medicine at Mount Sinai, New York, NY 10029, USA; claire.colas@gmail.com (C.C.); avner.schlessinger@mssm.edu (A.S.)

**Keywords:** acid resistance, AdiC, cancer metabolism, enterobacteria, l-arginine/agmatine transporter, l-type amino acid transporter, LAT1, melting temperature, thermostability

## Abstract

The l-arginine/agmatine transporter AdiC is a prokaryotic member of the SLC7 family, which enables pathogenic enterobacteria to survive the extremely acidic gastric environment. Wild-type AdiC from *Escherichia coli,* as well as its previously reported point mutants N22A and S26A, were overexpressed homologously and purified to homogeneity. A size-exclusion chromatography-based thermostability assay was used to determine the melting temperatures (*T*_m_s) of the purified AdiC variants in the absence and presence of the selected ligands l-arginine (Arg), agmatine, l-arginine methyl ester, and l-arginine amide. The resulting *T*_m_s indicated stabilization of AdiC variants upon ligand binding, in which *T*_m_s and ligand binding affinities correlated positively. Considering results from this and previous studies, we revisited the role of AdiC residue S26 in Arg binding and proposed interactions of the α-carboxylate group of Arg exclusively with amide groups of the AdiC backbone. In the context of substrate binding in the human SLC7 family member l-type amino acid transporter-1 (LAT1; SLC7A5), an analogous role of S66 in LAT1 to S26 in AdiC is discussed based on homology modeling and amino acid sequence analysis. Finally, we propose a binding mechanism for l-amino acid substrates to LATs from the SLC7 family.

## 1. Introduction

Extreme acid resistance systems enable pathogenic enterobacteria such as *Escherichia coli* strain O157:H7 to survive the strongly acidic environment of the stomach and subsequently colonize and infect the human gut. One of these systems is the arginine-dependent resistance system of *E. coli*, which consists of the decarboxylase AdiA and the antiporter AdiC [1]. The former decarboxylates l-arginine to agmatine, while the latter exchanges intracellular agmatine for extracellular l-arginine. This process leads to the removal of one proton from the cytoplasm in each turnover, thus maintaining the intracellular pH in a physiological range favorable for cell survival. AdiC belongs to the amino acid/polyamine/organocation (APC) transporter superfamily [2] and is a prokaryotic homolog of the human SLC7 family of amino acid transporters [3].

Six crystal structures of AdiC have been reported so far, which were elucidated in different states, and in the absence and presence of substrate, i.e., in the outward-open, substrate-free [4,5,6], outward-open, l-arginine- [7] and agmatine-bound [6], and outward-facing occluded l-arginine-bound states [8]. These three-dimensional (3D) structures are of great value for the understanding of the molecular working mechanism of AdiC, and of other APC superfamily transporters including mammalian SLC7 family members. Furthermore, and because structure determination of mammalian transporters is highly challenging, homology models of mammalian transporters based on structures of bacterial homologs are of great interest and help towards the understanding of transport function and the identification of new inhibitors and substrates. In recent years, homology modeling combined with structure-function studies or structure-based ligand discovery were reported for human and mammalian SLC7 family members, e.g., for the l-amino acid transporter-1 (LAT1; SLC7A5) [9,10,11,12,13,14,15,16] and -2 (LAT2; SLC7A8) [17,18,19,20].

**Figure 1:**
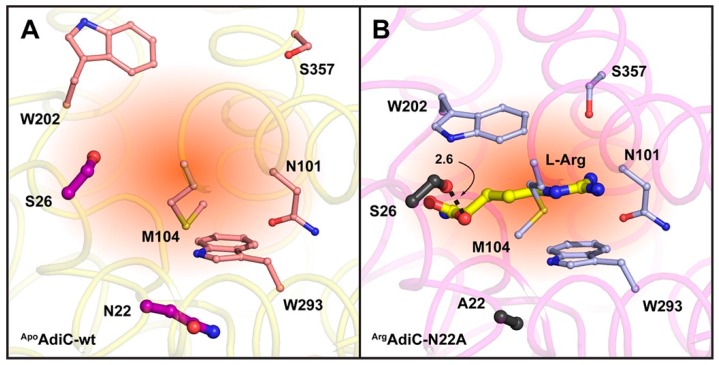
View into the substrate binding sites of the AdiC crystal structures in the outward-open, substrate-free (^apo^AdiC-wt; PDB ID code: 5J4I) (**A**); and outward-facing, occluded, l-arginine-bound states (^Arg^AdiC-N22A; PDB ID code: 3L1L) (**B**). Amino acid side chains located in the substrate binding pockets and reported to interact with substrates are displayed as sticks. The exception represents N22, which is located near the substrate binding site and was not reported to interact with substrates in the currently available crystal structures. The volumes of the substrate binding pockets (indicated and colored in dark orange) are different in the outward-open and outward-facing occluded states because of the different protein conformations and positions of residue W202. Residues N22 and S26, which are pertinent to the presented study, are colored in magenta and black in the ^apo^AdiC-wt and ^Arg^AdiC-N22A structures, respectively. The bound Arg molecule in the ^Arg^AdiC-N22A structure is colored in yellow. The ^apo^AdiC-wt and ^Arg^AdiC-N22A structures are shown as ribbons colored in light-yellow and light-magenta. Besides N22 and S26, amino acid side chains involved in substrate binding are colored in salmon and light-blue in the ^apo^AdiC-wt and ^Arg^AdiC-N22A structures, respectively. The hydrogen bond between S26 and the α-carboxylate group of Arg is indicated by a dotted line, as well as the distance in Å.

The effects of several single point mutants on the function of AdiC have been described in recent years [2,4,5,6,7,21,22]. Special attention was paid to the N22A (AdiC-N22A) [4,6,8] and S26A (AdiC-S26A) AdiC mutants [6,21,22]. Residues N22 and S26 are located near and in the substrate-binding pocket of AdiC, respectively [6] (Figure 1A). The N22A mutation improved the binding affinity for l-arginine by about six-fold over AdiC-wt [6,8], while retaining a transport activity similar to that of wild-type [4]. Although the molecular mechanism, which increases the affinity for Arg in AdiC-N22A, is unclear, the introduction of this mutation made possible the structure elucidation of AdiC with bound Arg. Based on this Arg-bound, outward-facing, occluded crystal structure of AdiC-N22A, it was proposed that S26 is involved in binding of the α-carboxylate group of Arg by donating one hydrogen bond (Figure 1B). However, this finding is inconsistent with recent results from two different laboratories, which show that S26 is neither essential for Arg binding [6] nor Arg transport [21,22], thus raising the pertinent question: Is the observed interaction between S26 and the α-carboxylate group of Arg specific to the mutant form AdiC-N22A but not the physiologically relevant wild-type form AdiC-wt [6]? As previously mentioned, an additional AdiC structure of a mutant (N101A) is available in the outward-open, l-arginine bound state [7]. The N101 residue is crucial for substrate binding (Figure 1B) [6,8]. Mutation N101A results in a defective AdiC protein that is unable to bind Arg [6,7] and with a dramatically decreased turnover rate compared with wild-type [7]. It is unclear how these two described functional features of a defective AdiC protein comply with the determination of an Arg-bound AdiC-N101A crystal structure.

**Figure 2:**
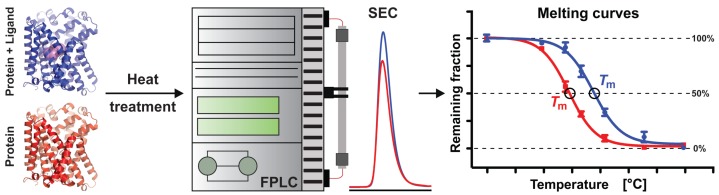
Workflow for *T*_m_ determination of ligand-free and -bound membrane protein using SEC. Purified membrane protein samples in the absence and presence of selected ligands are exposed to different temperatures for a defined time period and then subjected to SEC using a thermocycler and an FPLC, respectively. Peak heights in elution profiles enable the quantification of the fraction of membrane protein that remains intact after heat treatment. Plotting of remaining fractions versus temperatures results in melting curves from which *T*_m_ values are determined. Comparison of *T*_m_s allows determination of possible ligand-induced stabilization effects on purified membrane proteins, e.g., right shift of the blue melting curve (protein with ligand), indicating increased *T*_m_ compared to the red curve (protein without ligand). The flow chart was adapted from Mancusso et al. (2011) [24]. Membrane protein structures without and with bound ligand are coloured in red and blue (ligand in magenta), respectively.

In the context of protein crystallization for structure determination by X-ray crystallography, thermostability of purified membrane proteins in different detergents and at different pHs and ionic strengths is often assessed to identify optimal stabilizing conditions [23]. High protein thermostability is desirable, because it frequently translates into better crystals and thus into structures solved at higher resolutions. Importantly, and additionally to the previously mentioned parameters for protein stabilization, the addition of ligands and, in particular, of strong binders usually also increases protein thermostability. A simple method to assess thermostability of purified membrane proteins represents the determination of their melting temperature (*T*_m_) using size-exclusion chromatography (SEC) [24]. In the presented study, we have used a slightly adapted version of this method (Figure 2) to determine thermostability of wild-type AdiC and specific point mutants in the absence and presence of selected ligands. The obtained *T*_m_s were correlated to previously determined ligand inhibition constants (*K*_i_) and crystal structures, unveiling mechanistic insights into ligand binding of AdiC.

## 2. Results and Discussion

**Figure 3:**
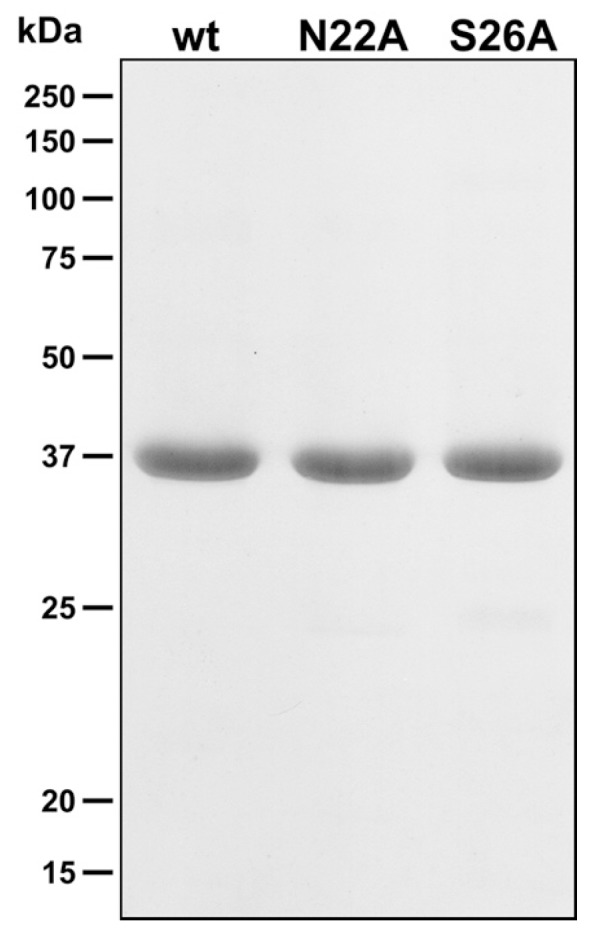
SDS-PAGE analysis of purified AdiC variants. A Coomassie brilliant-blue stained 13.5% SDS/polyacrylamide gel of wild-type AdiC (wt), and N22A and S26A AdiC mutants (5 µg of protein loaded per lane) is shown.

**Figure 4:**
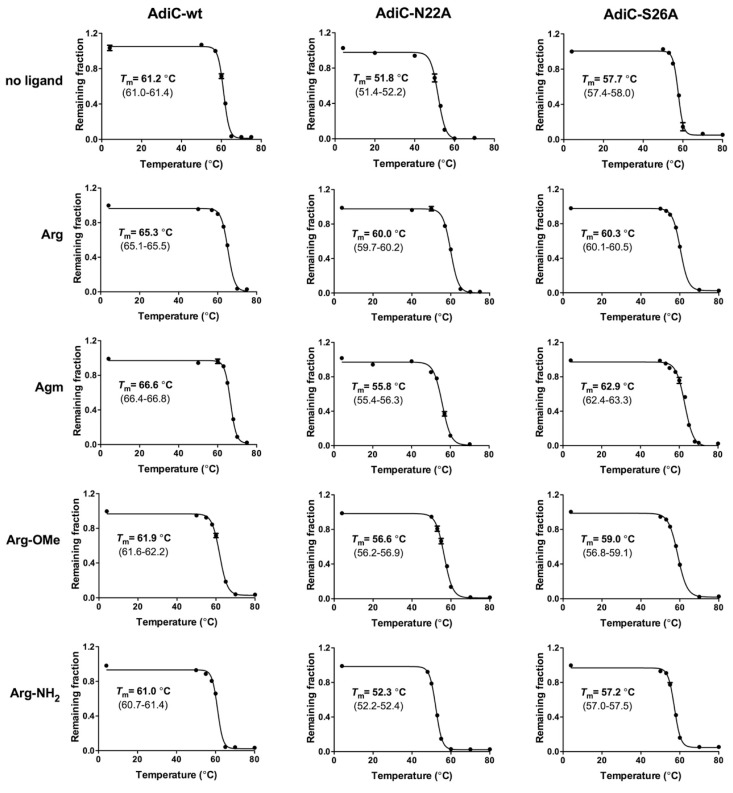
Thermostability curves and resulting melting temperatures of AdiC-wt, AdiC-N22A, and AdiC-S26A in the absence and presence of selected ligands. Ligands: l-arginine (Arg), agmatine (Agm), l-arginine methyl ester (Arg-OMe), and l-arginine amide (Arg-NH2). *T*_m_: melting temperature. The determined *T*_m_ values are from at least three independent experiments, each in triplicate, and 95% confidence interval values are indicated below *T*_m_s. Error bars represent SEM.

To explore the thermostabilities of AdiC variants in the absence and presence of selected ligands (200-fold molar excess), His-tagged AdiC-wt, AdiC-N22A, and AdiC-S26A proteins were overexpressed in *E. coli*, purified by nickel affinity chromatography, and depleted of their His-tag by human rhinovirus 3C protease treatment (see Materials and Methods for details). The high purity of the different purified AdiC proteins can be appreciated on the Coomassie Brilliant Blue stained SDS/polyacrylamide gel displayed in Figure 3. As is apparent from the gel, all AdiC variants ran at about 37 kDa by SDS-PAGE. The high protein purity and quality of the AdiC variants represented an excellent starting point for thermostability determination by SEC. Thermostabilities of the purified AdiC mutants were assessed and compared with those of AdiC-wt (Figure 4, row labeled *no ligand*). From the three AdiC variants, AdiC-wt showed, with 61.2 °C, the highest *T*_m_ compared to AdiC-N22A (*T*_m_ = 51.8 °C) and AdiC-S26A (*T*_m_ = 57.7 °C). In contrast, the thermostability of AdiC-N22A was remarkably compromised by a decrease of almost 10 °C compared to AdiC-wt. Clearly, introduction of the N22A and S26A single point mutations into AdiC reduced protein stability. Plausible explanations at the molecular level for the observed decreases in thermostabilities will most probably be possible once ligand-free crystal structures of these two AdiC mutants are available. Interestingly, addition of Arg significantly stabilized AdiC-N22A increasing the *T*_m_ by 8.2 °C (Figure 4, row *Arg*), whereas the *T*_m_s of AdiC-wt and AdiC-S26A increased less dramatically in the presence of Arg, i.e., by 4.1 °C and 2.6 °C, compared to AdiC-N22A. Considering that AdiC-wt is, with a *T*_m_ of 65.3 °C, the most stable AdiC variant in the presence of Arg, and considering one of the conceptual ideas from crystallography, namely that successful crystallization goes with high protein stability, innumerous crystallization trials failed in structure determination of AdiC-wt with bound Arg [6]. In contrast, co-crystallization of the less thermostable AdiC-N22A with Arg successfully yielded well-diffracting crystals and an Arg-bound AdiC-N22A structure in the outward-facing occluded state at 3 Å resolution [8]. Keeping in mind the results from thermostability, one might speculate that purified AdiC-N22A is present in the outward open conformation and that upon addition of Arg binding of this substrate induces a conformational change of the transporter into the outward-facing occluded state, which significantly stabilizes the complex (Δ*T*_m_ = 8.2 °C) and traps the ligand. Isothermal titration calorimetry (ITC) and scintillation proximity assay (SPA) data showed that the other AdiC main substrate, i.e., agmatine (Agm), binds with 2.6- and 3.2-fold higher affinities to AdiC-wt compared to Arg [6,25]. In line with this finding, the *T*_m_ of AdiC-wt with Agm was higher than with Arg (compare in Figure 4, rows *Arg* and *Agm*), indicating stronger stabilizing protein-ligand interactions. Furthermore, highly-ordered 3D crystals of Agm-bound AdiC-wt could be successfully obtained, and the structure in the Agm-bound, outward-open state was solved at 2.6 Å resolution [6], which supported the previously mentioned conceptual idea from crystallography. A similar increase of *T*_m_ as for AdiC-wt (Δ*T*_m_ = 5.4 °C) was also observed for AdiC-S26A (Δ*T*_m_ = 5.2 °C) in the presence of Agm, which suggests same Agm binding mechanisms and protein-ligand interactions (Figure 4). In stark contrast to AdiC-wt and AdiC-S26A, this trend was inverted for AdiC-N22A, i.e., Agm had a weaker thermostabilizing effect on the protein compared to Arg. This observation is in line with an 8.3-fold lower binding affinity for Agm compared to Arg in AdiC-N22A as previously determined by SPA [6], and thus with weaker intermolecular interactions between Agm and AdiC-N22A. Both, i.e., the inversion of affinities for Arg and Agm, and of thermostabilities in the presence of these substrates when compared to AdiC-wt, raise the critical question: Does the structure and function of AdiC-N22A reflect the physiological substrate binding mechanism of the wild-type transporter? Thermostabilization of AdiC-wt, AdiC-N22A, and AdiC-S26A for the Arg analogs l-arginine methyl ester (Arg-OMe) and l-arginine amide (Arg-NH_2_) was also explored by SEC (Figure 4, rows *Arg-OMe* and *Arg-NH*_2_). Thermostabilization between AdiC variants in the presence and absence of ligand, i.e., Δ*T*_m_, was for Arg-OMe and AdiC-N22A the highest (Δ*T*_m_ = 4.8 °C), followed by AdiC-S26A (Δ*T*_m_ = 1.3 °C) and AdiC-wt (Δ*T*_m_ = 0.7 °C). Thus, introduction of a methyl group and absence of the negative charge in Arg-OMe compared to Arg induced a loss of thermostability (Δ*T*_m_) of about twofold in AdiC-N22A and AdiC-S26A, and of about six-fold in AdiC-wt. For the latter, *T*_m_s with and without Arg-OMe were comparable, only displaying a neglectable Δ*T*_m_ of 0.7 °C and therefore indicating very weak binding of Arg-OMe to AdiC-wt. Thus, and based on the newly generated thermostability data, we conclude that Arg-OMe only had a significant stabilizing effect on AdiC-N22A, in contrast to AdiC-S26A and AdiC-wt in which thermostabilization was low and neglectable, respectively. In line with our previous observation, ligand binding to AdiC-N22A differs considerably from AdiC-wt, indicating that this AdiC mutant does not represent an appropriate model to study and understand the ligand binding mechanism of the physiologically relevant wild-type AdiC form. l-arginine amide (Arg-NH_2_) was previously used as an analogue of protonated Arg^2+^ [5,6,21,22]. All three AdiC variants showed comparable Δ*T*_m_ values in the presence and absence of Arg-NH_2_, suggesting no or very weak protein-ligand interactions and thus no ligand-binding-based protein stabilization.

**Figure 5:**
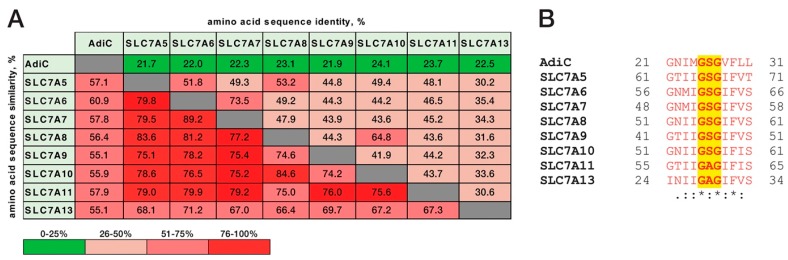
Amino acid sequence comparison between AdiC from *E. coli* and human l-amino acid transporters (LATs) from the SLC7 family. Identity and similarity values between different sequences are indicated in percentages and color scored. The Asc-2 (Slc7a12) and arpAT (Slc7a15) LATs were not considered in the sequence analysis, because their genes are not present or highly inactivated in primate genomes [26].

Based on the previously published, outward-facing, occluded l-arginine-bound AdiC-N22A structure, it was proposed that S26 plays a role in recognition and binding of the α-carboxylate group of the substrate Arg [8]. However, as previously mentioned, binding [6] and uptake studies [21,22] indicated no relevant role of S26 in Arg binding and transport. This finding is further supported by the here presented thermostability study. The structure of AdiC is being used for homology modeling of human LATs from the SLC7 family because of its acceptable amino acid sequence identity and similarity (Figure 5A), and high conservation of the binding site [3,9]. Interestingly, the majority of the human LATs from the SLC7 family also contain serine residues corresponding to S26 of AdiC and the flanking glycine residues (Figure 5B). The amide nitrogen of the glycine residue succeeding the serine residue in the helix-breaking GSG motif was shown to form a hydrogen bond with the α-carboxylate group of the Arg substrate in AdiC [8]. Notably, the mutation S26A in the AdiC region near the α-carboxylate group of Arg was reported not to influence Arg binding or transport [6,21,22], suggesting that protein backbone interactions with the α-carboxylate group of Arg are solely responsible for binding and are similar to the binding of the α-amino group of this amino acid and agmatine by main-chain carbonyl oxygen atoms from the AdiC protein [6,8]. This suggestion is further supported by the existence of the GAG instead of GSG sequence in the LATs SLC7A11 and SLC7A13 (Figure 5B).

**Figure 6:**
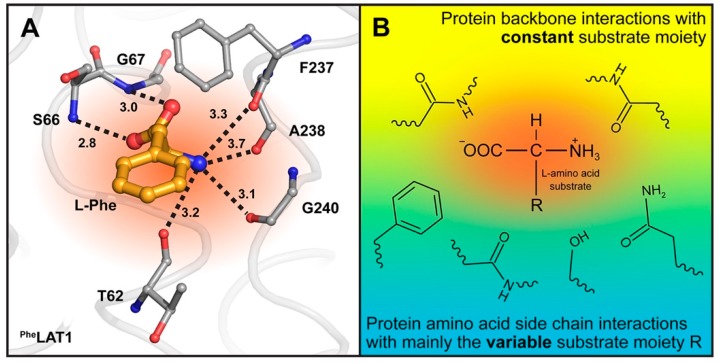
Homology model of human LAT1 with docked l-Phe substrate (^Phe^LAT1) and l-amino acid binding hypothesis for LATs. (**A**) View into the substrate binding site (colored in dark orange) of the AdiC structure-based human LAT1 homology model [9]. Oxygen and nitrogen atoms from carbonyl and amide groups of protein backbone amino acid residues (displayed as sticks and colored in grey) that are in hydrogen bond distance to the α-amino and α-carboxyl groups of the substrate l-Phe (colored in gold) are indicated as dotted lines. Interatomic distances are indicated, and numbers correspond to Å. The ^Phe^LAT1 model is shown as ribbon colored in light-grey. (**B**) Potential binding mechanism of l-amino acid substrates to LATs from the SLC7 family. Based on the LAT1 homology model (**A**) [9] and the available AdiC structures with bound substrates [6,8], protein backbone interactions via carbonyl and amide groups with the α-amino and α-carboxyl group of the amino acid substrates are proposed. For interactions with the variable R-group of amino acid substrates, one backbone interaction and arbitrary amino acid side chains are displayed.

Accurate modeling of the human SLC7 family members is required for understanding the substrate specificity determinants in this family, as well as for rational drug design against these emerging therapeutic targets [3,13]. For example, the l-type amino acid transporter LAT1 (SLC7A5) is important for transporting drugs across the blood-brain-barrier, as well as for providing amino acid nutrients in cancer [27,28]. Interestingly, homology models of human LAT1 based on the AdiC structure as template support the findings of these data regarding S66, which corresponds to S26 in AdiC: The occluded LAT1 model [9,29] with its substrate l-Phe docked in the binding pocket shows that the α-carboxylate group of the amino acid ligand is coordinated through hydrogen bonds with backbone atoms of the binding site residues S66 and G67 (Figure 6A). While homology models cannot reliably assign side chains of amino acids, modeling the side chain of S66 to face the amino acid substrate does not improve the model’s ability to enrich for ligands compared to other compounds [12,14], supporting the analogous role of S26 in AdiC and S66 in LAT1. Furthermore, and as previously mentioned, the GSG motif in AdiC and LAT1 is substituted with GAG in the cystine/glutamate transporter SLC7A11 (xCT) and the aspartate/glutamate transporter SLC7A13 (Figure 5B), where these transporters handle amino acid substrates despite lacking the serine residue in this position. Finally, the finding that the α-carboxylate, as well as the α-amino groups of the amino acid substrates Phe and Arg of LAT1 and AdiC, are recognized and bound by the protein backbone, and the variable parts of the mentioned substrates mainly by protein amino acid side chains [6,8,9], suggests an evolutionary hypothesis. During evolution of such amino acid transporters, it seems to have been beneficial to bind the constant and variable parts of substrates by protein backbone and mainly amino acid side chains, respectively. The former brings constancy, being relatively insensitive to mutations, while the latter can vary by mutagenesis through evolution to allow binding of different amino acid substrates (Figure 6B).

## 3. Materials and Methods

### 3.1. Cloning and Overexpression of AdiC

Cloning of the AdiC gene into the pZUDF21 vector was performed as described previously [6,30] and resulted in a recombinant AdiC protein with a human rhinovirus 3C protease cleavage site followed by a deca-His tag at the C terminus. For generation of AdiC mutants, the QuikChange Lightning Multi Site-Directed Mutagenesis Kit (Agilent) was used. Overexpression of AdiC variants in *E. coli* was performed as described previously [6,30].

### 3.2. Membrane Preparation and Purification of AdiC

Membranes containing overexpressed recombinant AdiC protein were prepared from bacterial cells as described previously [6,30]. For purification, one aliquot of membrane suspension corresponding to 1 L of cell culture was solubilized for 2 h at 4 °C on a rotational shaker in 20 mM Tris-HCl (pH 8.0), 300 mM NaCl, 10% (*v*/*v*) glycerol, 1.5% (*w*/*v*) *n*-dodecyl-*β*-d-maltopyranoside (DDM; Glycon Biochemicals GmbH, Luckenwalde, Germany) (*V*_tot_ = 7 mL). After ultracentrifugation (100,000× *g* for 1 h at 4 °C), the supernatant was diluted twofold in the purification buffer (20 mM Tris-HCl (pH 8.0), 300 mM NaCl, 10% glycerol, 0.04% (*w*/*v*) DDM, 5 mM l-histidine) and incubated with 0.5 mL (bed volume) pre-equilibrated Ni-NTA Superflow resin (Qiagen, Valencia, CA, USA) for 2 h at 4 °C on a rotational shaker (metal affinity chromatography). The resin was then transferred into a column and washed three times with 5 mL of purification buffer, which was followed by an additional washing step with 3 mL of the same buffer without l-histidine by gravity flow. The His-tag on AdiC was removed by on-column cleavage on a rotational shaker for 18 h at 4 °C by addition of 200 µg of HRV3C protease (BioVision, Milpitas, CA, USA). Finally, the cleaved AdiC was eluted by centrifugation (3000× *g* for 15 s at 4 °C), and remaining HRV3C was removed by an additional incubation of the eluate with 100 µL (bed volume) pre-equilibrated Ni-NTA Superflow resin on a rotational shaker for 15 min at 4 °C followed by a subsequent filtration to remove the resin. For protein concentration determination, the bicinchoninic acid assay (BCA; Pierce, Thermo Scientific) was used.

### 3.3. Determination of AdiC Thermostability

Thermostability analysis of AdiC variants was performed as described previously [24]. To test the effect of ligands on the thermostability of AdiC variants, purified samples in aliquots of 35 µg (70 µL total volume and ~11 µM AdiC concentration) were first incubated with the ligands (2 mM; corresponding to 200-fold molar excess) on ice for 10 min. Next, the samples were incubated at different temperatures for 10 min using a Labcycler gradient PCR machine (SensoQuest GmbH, Göttingen, Germany). After a short centrifugation to remove eventual aggregates (18,000× *g* for 30 s at room temperature), the supernatant was loaded on a Superdex 200 5/150 GL column (GE Healthcare) installed on an ÄKTA Purifier system (GE Healthcare). Prior to injection, the column was equilibrated with 1.5 column volumes of SEC-buffer (20 mM Tris-HCl (pH 8.0), 150 mM NaCl, 0.04% (*w*/*v*) DDM, and 2 mM of tested ligand). The SEC peak absorbance values at 280 nm of intact AdiC remaining after denaturation and of untreated sample (reference for normalization; sample kept on ice) were measured and used to calculate the remaining fraction. Remaining fractions against incubation temperatures were plotted (Figure 2) and melting temperatures (*T*_m_s) were calculated by applying the Boltzmann sigmoidal equation in Prism5 (GraphPad Software, La Jolla, CA, USA).

### 3.4. Amino Acid Sequence Analysis

The amino acid sequence identity and similarity analyses were carried out with LALIGN (https://embnet.vital-it.ch/software/LALIGN_form.html) by applying local alignment method using default settings, i.e., BLOSUM50 scoring matrix, 3 as number of reported sub-alignments, 10.0 as e-value threshold, -12 as opening gap penalty, and -2 as extending gap penalty. For the multiple sequence alignment of the human SLC7 family members (SLC7A5-11 and SLC7A13) and their bacterial homologue AdiC, Clustal Omega [31] was used. The UniProtKB/Swiss-Prot entry codes of the used proteins are: P60061 (AdiC), Q01650 (SCL7A5), Q92536 (SLC7A6), Q9UM01 (SLC7A7), Q9UHI5 (SLC7A8), P82251 (SLC7A9), Q9NS82 (SLC7A10), Q9UPY5 (SLC7A11), and Q8TCU3 (SLC7A13).

### 3.5. Homology Models and Ligand Docking

We show a previously published homology model of LAT1 bound to phenylalanine, which was built based on the outward-facing occluded conformation of AdiC [9] (PDB ID code: 3L1L). Briefly, to construct homology models of LAT1, we generated a multiple sequence alignment containing the human SLC7 members and the AdiC structure using PROMALS3D [32] (default settings), which was subsequently refined based on structural considerations. Initial LAT1 models were built using MODELLER [33] and refined using SCWRL4 [34]. The final model was selected based on its ability to distinguish between known ligands and decoy compounds, capturing its relevance for describing protein-ligand interactions.

## 4. Conclusions

In this study, we determined the melting temperatures of AdiC-wt, AdiC-N22A, and AdiC-S26A in the absence and presence of the ligands Arg, Agm, Arg-OMe, and Arg-NH_2_. Stabilization of AdiC variants through binding of specific ligands (as determined by SEC; Figure 2) correlated with increased binding affinities to these molecules as previously determined by ITC [2,21,25] and SPA [6,35,36]. Our observations on the binding mechanism of the α-carboxylate group of Arg in AdiC were extrapolated to the SLC7 family and supported by the homology model of human LAT1. From a methodological point of view, the determination of melting temperatures by SEC analysis of membrane proteins in the absence and presence of ligands represents an attractive approach to determine qualitatively ligand binding and protein stabilization in a label-free way. Finally, our study indicates that particular care has to be taken when working with mutants, because obtained results and deduced working mechanisms, e.g., substrate binding, might not fully reflect the physiologically important wild-type protein.

## References

[1] Foster J.W. (2004). *Escherichia coli* acid resistance: Tales of an amateur acidophile. Nat. Rev. Microbiol..

[2] Casagrande F., Ratera M., Schenk A.D., Chami M., Valencia E., Lopez J.M., Torrents D., Engel A., Palacin M., Fotiadis D. (2008). Projection structure of a member of the amino acid/polyamine/organocation transporter superfamily. J. Biol. Chem..

[3] Fotiadis D., Kanai Y., Palacin M. (2013). The SLC3 and SLC7 families of amino acid transporters. Mol. Asp. Med..

[4] Gao X., Lu F., Zhou L., Dang S., Sun L., Li X., Wang J., Shi Y. (2009). Structure and mechanism of an amino acid antiporter. Science.

[5] Fang Y., Jayaram H., Shane T., Kolmakova-Partensky L., Wu F., Williams C., Xiong Y., Miller C. (2009). Structure of a prokaryotic virtual proton pump at 3.2 Å resolution. Nature.

[6] Ilgü H., Jeckelmann J.-M., Gapsys V., Ucurum Z., de Groot B.L., Fotiadis D. (2016). Insights into the molecular basis for substrate binding and specificity of the wild-type L-arginine/agmatine antiporter AdiC. Proc. Natl. Acad. Sci. USA.

[7] Kowalczyk L., Ratera M., Paladino A., Bartoccioni P., Errasti-Murugarren E., Valencia E., Portella G., Bial S., Zorzano A., Fita I. (2011). Molecular basis of substrate-induced permeation by an amino acid antiporter. Proc. Natl. Acad. Sci. USA.

[8] Gao X., Zhou L., Jiao X., Lu F., Yan C., Zeng X., Wang J., Shi Y. (2010). Mechanism of substrate recognition and transport by an amino acid antiporter. Nature.

[9] Geier E.G., Schlessinger A., Fan H., Gable J.E., Irwin J.J., Sali A., Giacomini K.M. (2013). Structure-based ligand discovery for the large-neutral amino acid transporter 1, LAT-1. Proc. Natl. Acad. Sci. USA.

[10] Dickens D., Webb S.D., Antonyuk S., Giannoudis A., Owen A., Radisch S., Hasnain S.S., Pirmohamed M. (2013). Transport of gabapentin by LAT1 (SLC7A5). Biochem. Pharmacol..

[11] Napolitano L., Scalise M., Galluccio M., Pochini L., Albanese L.M., Indiveri C. (2015). LAT1 is the transport competent unit of the LAT1/CD98 heterodimeric amino acid transporter. Int. J. Biochem. Cell Biol..

[12] Augustyn E., Finke K., Zur A.A., Hansen L., Heeren N., Chien H.C., Lin L., Giacomini K.M., Colas C., Schlessinger A. (2016). LAT-1 activity of meta-substituted phenylalanine and tyrosine analogs. Bioorg. Med. Chem. Lett..

[13] Colas C., Ung P.M., Schlessinger A. (2016). SLC transporters: Structure, function, and drug discovery. MedChemComm.

[14] Zur A.A., Chien H.C., Augustyn E., Flint A., Heeren N., Finke K., Hernandez C., Hansen L., Miller S., Lin L. (2016). LAT1 activity of carboxylic acid bioisosteres: Evaluation of hydroxamic acids as substrates. Bioorg. Med. Chem. Lett..

[15] Napolitano L., Galluccio M., Scalise M., Parravicini C., Palazzolo L., Eberini I., Indiveri C. (2017). Novel insights into the transport mechanism of the human amino acid transporter LAT1 (SLC7A5). Probing critical residues for substrate translocation. Biochim. Biophys. Acta.

[16] Napolitano L., Scalise M., Koyioni M., Koutentis P., Catto M., Eberini I., Parravicini C., Palazzolo L., Pisani L., Galluccio M. (2017). Potent inhibitors of human LAT1 (SLC7A5) transporter based on dithiazole and dithiazine compounds for development of anticancer drugs. Biochem. Pharmacol..

[17] Rosell A., Meury M., Alvarez-Marimon E., Costa M., Perez-Cano L., Zorzano A., Fernandez-Recio J., Palacin M., Fotiadis D. (2014). Structural bases for the interaction and stabilization of the human amino acid transporter LAT2 with its ancillary protein 4F2hc. Proc. Natl. Acad. Sci. USA.

[18] Hinz K.M., Meyer K., Kinne A., Schulein R., Kohrle J., Krause G. (2015). Structural insights into thyroid hormone transport mechanisms of the L-type amino acid transporter 2. Mol. Endocrinol..

[19] Hinz K.M., Neef D., Rutz C., Furkert J., Kohrle J., Schulein R., Krause G. (2017). Molecular features of the L-type amino acid transporter 2 determine different import and export profiles for thyroid hormones and amino acids. Mol. Cell. Endocrinol..

[20] Krause G., Hinz K.M. (2017). Thyroid hormone transport across L-type amino acid transporters: What can molecular modelling tell us?. Mol. Cell. Endocrinol..

[21] Tsai M.F., Fang Y., Miller C. (2012). Sided functions of an arginine-agmatine antiporter oriented in liposomes. Biochemistry.

[22] Tsai M.F., Miller C. (2013). Substrate selectivity in arginine-dependent acid resistance in enteric bacteria. Proc. Natl. Acad. Sci. USA.

[23] Magnani F., Serrano-Vega M.J., Shibata Y., Abdul-Hussein S., Lebon G., Miller-Gallacher J., Singhal A., Strege A., Thomas J.A., Tate C.G. (2016). A mutagenesis and screening strategy to generate optimally thermostabilized membrane proteins for structural studies. Nat. Protoc..

[24] Mancusso R., Karpowich N.K., Czyzewski B.K., Wang D.N. (2011). Simple screening method for improving membrane protein thermostability. Methods.

[25] Fang Y., Kolmakova-Partensky L., Miller C. (2007). A bacterial arginine-agmatine exchange transporter involved in extreme acid resistance. J. Biol. Chem..

[26] Fernandez E., Torrents D., Zorzano A., Palacin M., Chillaron J. (2005). Identification and functional characterization of a novel low affinity aromatic-preferring amino acid transporter (arpAT). One of the few proteins silenced during primate evolution. J. Biol. Chem..

[27] Nicklin P., Bergman P., Zhang B., Triantafellow E., Wang H., Nyfeler B., Yang H., Hild M., Kung C., Wilson C. (2009). Bidirectional transport of amino acids regulates mTOR and autophagy. Cell.

[28] Altman B.J., Stine Z.E., Dang C.V. (2016). From Krebs to clinic: Glutamine metabolism to cancer therapy. Nat. Rev. Cancer.

[29] Schlessinger A., Khuri N., Giacomini K.M., Sali A. (2013). Molecular modeling and ligand docking for solute carrier (SLC) transporters. Curr. Top. Med. Chem..

[30] Ilgü H., Jeckelmann J.M., Gachet M.S., Boggavarapu R., Ucurum Z., Gertsch J., Fotiadis D. (2014). Variation of the detergent-binding capacity and phospholipid content of membrane proteins when purified in different detergents. Biophys. J..

[31] McWilliam H., Li W., Uludag M., Squizzato S., Park Y.M., Buso N., Cowley A.P., Lopez R. (2013). Analysis Tool Web Services from the EMBL-EBI. Nucleic Acids Res..

[32] Pei J., Kim B.H., Grishin N.V. (2008). PROMALS3D: A tool for multiple protein sequence and structure alignments. Nucleic Acids Res..

[33] Eswar N., Webb B., Marti-Renom M.A., Madhusudhan M.S., Eramian D., Shen M.Y., Pieper U., Sali A. (2006). Comparative protein structure modeling using Modeller. Curr. Protoc. Bioinform..

[34] Krivov G.G., Shapovalov M.V., Dunbrack R.L. (2009). Improved prediction of protein side-chain conformations with SCWRL4. Proteins.

[35] Meury M., Harder D., Ucurum Z., Boggavarapu R., Jeckelmann J.-M., Fotiadis D. (2011). Structure determination of channel and transport proteins by high-resolution microscopy techniques. Biol. Chem..

[36] Harder D., Fotiadis D. (2012). Measuring substrate binding and affinity of purified membrane transport proteins using the scintillation proximity assay. Nat. Protoc..

